# Asparagine Synthetase Gene *OsASN2* Is Crucial for Rice Seed Development and Germination

**DOI:** 10.3390/plants14131999

**Published:** 2025-06-30

**Authors:** Rui Hu, Kaiming Liang, Xiangyu Hu, Meijuan Li, Qunhuan Ye, Yuanhong Yin, Cai Tang, Xinyu Wang, Youqiang Fu, Junfeng Pan, Mingyong Zhang, Xuhua Zhong

**Affiliations:** 1 Rice Research Institute, Guangdong Academy of Agricultural Sciences/Key Laboratory of Genetics and Breeding of High Quality Rice in Southern China (Co-Construction by Ministry and Province), Ministry of Agriculture and Rural Affairs/Guangdong Key Laboratory of Rice Science and Technology/Guangdong Rice Engineering Laboratory, Guangzhou 510640, China; hurui@gdaas.cn (R.H.);; 2 Guangdong Provincial Key Laboratory of Applied Botany, South China Botanical Garden, Chinese Academy of Sciences, Guangzhou 510650, China; zhangmy@scbg.ac.cn

**Keywords:** asparagine, amino acid metabolism, endosperm development, rice

## Abstract

Seed development plays a critical role in determining both crop yield and grain quality in rice. As a key nutrient storage organ, the rice endosperm development not only contributes to grain filling but also plays an essential role during the early stages of seed germination. Amino acid metabolism is active during the process of seed development and seed germination. Asparagine is a primary amino acid responsible for long-distance organic nitrogen transport in plants. Asparagine synthetase catalyzes the synthesis of asparagine from aspartate and glutamine. In this study, CRISPR/Cas9-mediated knockout mutants of the *OsASN2* gene of rice were generated. Homozygous mutants exhibited complete failure of seed germination, and heterozygotes could not produce homozygous offspring. Endosperm development of homozygous mutant seeds showed severe defects. Additionally, interacting protein screening combined with pull-down and co-immunoprecipitation (Co-IP) assays confirmed that OsASN2 physically interacted with pyruvate phosphate dikinase OsPPDKB, the mutants of which showed impaired endosperm development. These findings collectively indicate that *OsASN2* plays a critical role in seed development and germination in rice.

## 1. Introduction

The increasing global demand for high-quality rice has made the improvement of grain quality a key focus in rice genetic breeding. Development of seeds determines the crop yield and grain quality. The rice endosperm, as the major edible part of the rice seed, is the primary site for storage compound deposition, and is most related to rice yield and quality traits, such as milling, cooking, and eating qualities. Therefore, studying cereal endosperms will contribute to the breeding of cereal crops, resulting in improved yields as well as improved cooking and nutritional qualities [1].

In rice, a model cereal crop, endosperm development proceeds four sequential phases: (1) coenocytic nuclear division (0–2 days after pollination (DAP)), (2) cellularization (3–5 DAP), (3) aleurone/starchy endosperm differentiation (6–9 DAP), and (4) storage product accumulation (6–20 DAP), with the latter two stages temporally overlapping. Storage product accumulation continues through 20 DAP, during which starch granules gradually fill the cellular space of the starchy endosperm [1,2]. Current research has identified some gene families governing rice endosperm cell development, normal grain filling, and maturation. The PRC2 complex mediates chromatin epigenetic modifications and regulates developmental phase transitions in plants. Mutations in PRC2-encoding genes lead to delayed or failed cellularization. The CRISPR/Cas9-derived mutant *OsFIE2* exhibited impaired cellularization of the endosperm, which suggests that *OsFIE2* is indispensable for early seed development as a positive regulator of cellularization [3]. Cheng et al. found that OsEMF2a-PRC2-mediated H3K27me3 was necessary for endosperm cellularization and genomic imprinting in rice [4]. Endosperm development involves tightly regulated cell cycle control during syncytium formation, cellularization, and endoreduplication. Critical cell cycle regulators, including *CycB1;1*, *KRP1*, and *ENL1*, influence cellularization processes [5,6,7]. The accumulation of starch plays a decisive role in rice yield and quality formation. Current research primarily focuses on genes associated with sucrose and starch synthesis and transport. For instance, mutation of gene *OsCIN2* (*GIF1*), which encodes the cell wall invertase, which in turn hydrolyzes sucrose, results in incomplete grain filling [8]. Similarly, RNA interference-mediated suppression of the sucrose transporter gene *OsSUT1* causes abnormal grain filling [9]. Mutations in sucrose transporter genes *OsSWEET11* and *OsSWEET15* also leads to unfilled grains [10,11]. The initiation of starch synthesis by starch synthase is also tightly regulated. OsLESV can bind directly to starch via its C-terminal tryptophan-rich domain, and interacts with the starch-binding protein FLO6. Loss of function in either gene disrupts the targeting of isoamylase 1 (ISA1) to starch granules [12].

Successful germination in rice is facilitated by the breakdown of starch in the endosperm, leading to the accumulation of free sugars, such as glucose, sucrose, and fructose. Genes involved in sugar utilization have been shown to be important in germination. Trehalose-6-phosphate phosphatase (OsTPP7) enhances starch degradation. IR64 with the absence of *OsTPP7* barely germinates and has a limited coleoptile elongation under submergence [13]. Lipid transporter genes are also related to seed germination. Suppressing the expression of *OsLTP36*, which encodes a lipid transporter, results in a decreased germination rate [14]. Hormones play important roles in regulating seed germination, particularly abscisic acid (ABA) and gibberellin (GA). ABA inhibits germination. Seeds germinate when the endogenous ABA level decreases in the imbibing seed. Nine-cis-epoxycarotenoid dioxygenase (NCED), cleaving the cis-isomers of violaxanthin and neoxanthin to xanthoxin, has been reported to be the rate-limiting step of ABA biosynthesis. The enhanced germination phenotype is frequently associated with the downregulation of *OsNCEDs* [15]. In contrast, downregulation of the ABA-degrading genes (*OsABA8ox1*, *OsABA8ox2*, and *OsABA8ox3*) inhibits seed germination [16]. GA plays an antagonistic role to ABA in the regulation of seed germination. *OsGA3ox2*, encoding a GA synthesis enzyme, is important for α-amylase induction during rice seed germination [17]. Beyond these genes, many genes related to phytohormone metabolism and signal transduction have been found to be important in germination.

Many studies have shown that amino acids are metabolized actively during plant seed development and germination [18,19]. During seed development, organic nitrogen is transported from the leaves to the developing seeds. Asparagine (Asn) and glutamine (Gln) are the main forms of amino acid transport. Therefore, the roles of genes related to Asn biosynthesis and metabolism in seed development are worthy of in-depth investigation. Asparagine synthetase catalyzes the synthesis of asparagine (Asp). This enzyme is encoded by *ASN* genes. The question of how the *ASN* genes in rice influence seed development is an interesting one to be addressed. When searching for mutants in the T-DNA insertion databases, we did not find any mutants of *OsASN2*. In this study, we aimed to investigate the roles of *OsASN2* in the growth and development of rice. Therefore, CRISPR/Cas9-mediated knockout mutants of *OsASN2* in rice were generated to study the roles of *OsASN2* in rice development.

## 2. Materials and Methods

### 2.1. Creation and Detection of OsASN2

The CRISPR/Cas9 genome editing system, pioneered by Ma et al. [20], was utilized to create mutants of *OsASN2* (*LOC_Os06g15420* in RGAP and *Os06g0265000* in RAP-DB). Specifically, the anchor sequences, 5′-gcgggatagctcgatgatgcggg-3′ and 5′-gatcaacaatagccaaccgctgg-3′, were introduced into tRNA intron and sgRNA expression cassettes, driven by the *U3* promoter. To avoid the off-target effect, the anchor sequence was submitted to the off-target prediction database (http://skl.scau.edu.cn/offtarget/, accessed on 11 April 2025) to detect potential off-target sites. An off-target sequence will have more than two mismatches compared with the anchor sequence. The anchor sequences were precisely targeted to the first and second exons of *OsASN2*. This construct was introduced into the *Agrobacterium tumefaciens* strain *EHA*105, which was then transformed into ZH11 by *Agrobacterium*-mediated transformation. Transgenic calli were selected by 50 mg/L hygromycin [21]. To validate the editing events, PCR amplification was performed using primers designed to amplify the genomic region flanking the anchor sequence. The resulting PCR products were then sequenced and analyzed with the aid of DSDecodeM (http://skl.scau.edu.cn/dsdecode/, accessed on 11 April 2025) [20] to get the frameshift mutation on *OsASN2* coding sequence. When homozygotes were obtained, PCR was performed to detect the predicted off-target genes to ensure they were not edited.

### 2.2. Seed Germination and 2,3,5-Triphenyltetrazolium Chloride (TTC) Stain

2,3,5-triphenyltetrazolium chloride was dissolved in phosphate buffer (pH 7.0) to prepare a 1% (*w*/*v*) TTC staining solution. The seeds were pre-imbibed in sterilized water for 12 h, then the water in the surface of seeds was absorbed with filter paper. Seeds were immersed in the TTC staining solution and incubated at 30 °C for 2 h. The reaction was terminated by rinsing the stained seeds three times with distilled water. The stained seeds were observed under the stereomicroscope SteREO Lumar V12 (Zeiss, Oberkochen, Germany).

### 2.3. Scanning Electron Microscopy (SEM)

Transverse sections of rice seeds were fixed in glutaraldehyde fixative solution for over 24 h. The fixed samples were rinsed three times in 0.1 M phosphate-buffered saline (PBS), followed by dehydration through a graded ethanol series (pre-chilled) with 15 min intervals at each concentration. Subsequently, samples were infiltrated with tert-butanol three times (10 min per step), ensuring complete submersion in the final step. The samples were frozen at −20 °C for at least 30 min and subsequently subjected to freeze-drying in a vacuum freeze dryer. The dried samples were mounted on stubs using conductive adhesive, and a gold–palladium (Au–Pd) coating was applied via ion sputtering by EM ACE200 (Leica, Wetzlar, Germany). Images were captured with the scanning electron microscope SU-8010 (Hitachi, Tokyo, Japan).

### 2.4. Quantitative Real-Time RT-PCR (qPCR)

The isolation of total RNA was conducted with a HiPure Plant RNA Mini Kit (Magen, Guangzhou, China) as the manufacturer’s guidelines. Subsequently, the synthesis of cDNA was performed utilizing the EasyScript One-Step gDNA Removal and cDNA Synthesis SuperMix (TransGen Biotech, Beijing, China) to ensure high fidelity. For monitoring gene expression, qPCR was performed, employing *OsUBC* (*LOC_Os02g42314.2*) as the reference gene, as recommended by Jain et al. [22]. The qPCR procedure was executed in the presence of the double-stranded DNA-specific dye SYBR Green I (SYBR^®^ Premix Ex Taq GC, Takara, Kyoto, Japan), and real-time monitoring was conducted on the Roche LightCycler 480 system (Roche, Basel, Switzerland) to ensure precision and accuracy.

### 2.5. Detection of Interaction of OsASN2 and OsPPDKB by Immunoprecipitation-Mass Spectrometry (IP-MS) Screen, Pull-Down, and Co-Immunoprecipitation (Co-IP) Assay

To get *OsASN2-GFP* infusion constructs, the *OsASN2* coding sequence fragment was inserted downstream of the *35S* promoter of the *pBWA(V)HS* vector (from Biorun Bio-technology, Wuhan, China), to form an OsASN2-GFP infusion protein. This *OsASN2-GFP* construct was transformed into ZH11 by *Agrobacterium*-mediated transformation. Then, transgenic plants were used to extract the proteins. Transgenic *35S*: *GFP* plants were used as controls. The soluble protein extract was incubated with anti-GFP beads to capture OsASN2-GFP fusion proteins along with interacting proteins. Protein–antibody complexes were eluted to remove non-specific bindings. The eluted protein complexes were then subjected to liquid chromatography-tandem mass spectrometry analysis. The mass spectrometry data were acquired using a Vanquish NEO (ThermoFisher, Waltham, MA, USA) liquid chromatography system coupled with an Orbitrap Astral (ThermoFisher, Waltham, MA, USA) high-resolution mass spectrometer.

For the pull-down assay, an *OsASN2* coding sequence fragment was inserted into pMAL-c5x to form an MBP-OsASN2 infusion protein, and *OsPPDKB* was inserted into pET-28a to form a His-OsPPDKB infusion protein. These plasmids were transformed into BL21 and induced by 0.5 mM IPTG. For MBP pull-down assay, resin-bound MBP or MBP-OsASN2 proteins were mixed with His-OsPPDKB at 4 °C overnight. The elution was detected by immunoblot analysis using anti-His and anti-MBP antibodies.

For the Co-IP assay, *OsPPDKB-MYC* was constructed, *OsPPDKB* cDNA fragment was inserted downstream of the *35S* promoter of the *pBWA(V)pan580* vector (from Biorun Bio-technology, Wuhan, China). The GFP or OsASN2-GFP plasmids was transiently co-transformed into rice protoplasts with OsPPDKB-MYC, and Co-IP experiments using GFP-Trap were performed 12 h after transformation. Immunoblots were executed with anti-GFP antibodies to detect OsASN2 and with anti-MYC to detect OsPPDKB.

## 3. Results

### 3.1. The Mutation of OsASN2 Led to Developmental Defects in Rice

DNA sequencing confirmed that the *OsASN2* gene had a 3213 bp-long coding sequence, corresponding to a protein of 1070 amino acid residues. OsASN2 showed 72.8% and 80.6% protein identities with *Arabidopsis* ASN1 and ASN2, respectively.

**Figure 1:**
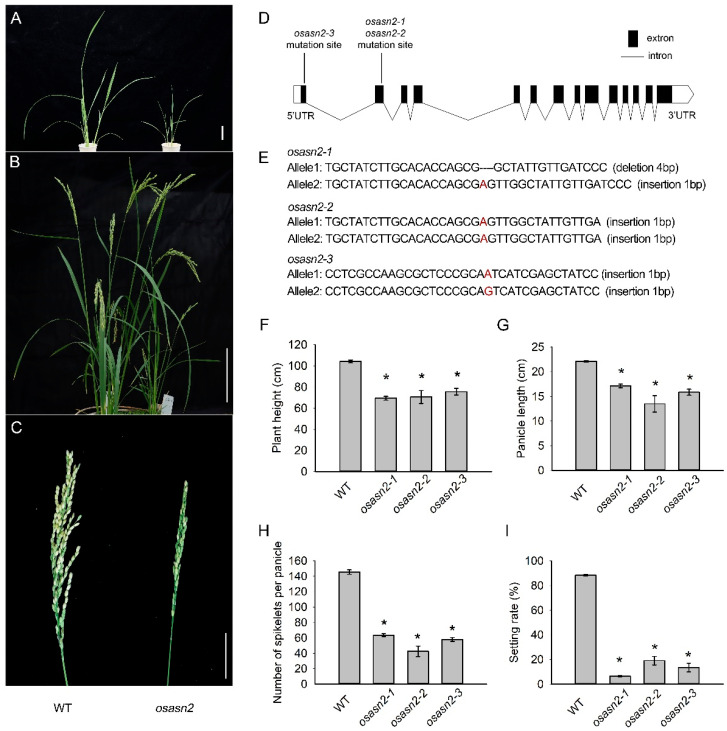
*OsASN2* knockout mutants showed developmental defects. (**A**,**B**) Phenotypes of the *osasn2* plants at vegetative stage and reproductive stage, respectively. Bar is 5 cm in A and 20 cm in B. (**C**) Panicle of *OsASN2*. bar = 5 cm. (**D**,**E**) *osasn2* was generated by the CRISPR/Cas9 system. The edited sequences of three represented lines are displayed. The dotted line represents deletion and the red base represents insertion. (**F**–**I**) Plant height, length of panicle, number of spikelets per panicle and seed setting rate of *osasn2*. Asterisks above the bars indicate significant differences between the *osasn2* and wild type (WT) plants (Student’s *t*-test, *p* < 0.05).

To identify the role of *OsASN2* in plant growth, knockout mutants of *OsASN2* were generated by the CRISPR/Cas9 system. PCR amplification was performed to get transgenic plants with frameshift mutations of *OsASN2*. Homozygous mutant plants of *OsASN2* were successfully obtained in T_0_ transgenic plants. The growth of *OsASN2* mutants was impaired in both the vegetative and reproductive stages (Figure 1A,B). Notably, the panicles of *osasn2* were shorter than those of the wild type (WT) (Figure 1C). Three representative lines were used for phenotype analysis at the mature stage (Figure 1D,E). The *osasn2-1* line had a 4 bp deletion and a 1 bp insertion in two chromosomes within the second exon of *OsASN2*, resulting in different mutations in two sister chromosomes. The *osasn2-2* line had the same 1 bp insertion in both chromosomes of the second exon of *OsASN2*. The *osasn2-3* line displayed a 1 bp insertion in both chromosomes in the first exon of *OsASN2*. The plant height, panicle length, number of spikelets per panicle, and setting rate of the mutants were significantly lower than those of the WT (Figure 1F–I). None of the heterozygous plants displayed these phenotypes in three independent transgenic events. Panicles of heterozygous mutants and WT plants are shown in Figure S1.

**Figure 2:**
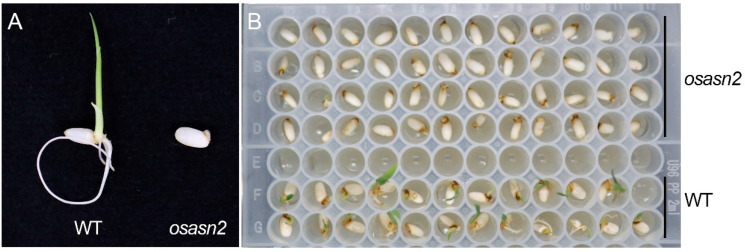
The seeds of *osasn2* did not germinate. Seeds were sown on 1/2 MS medium with sucrose. Five days’ seeds are pictured in (**A**) and 4 days in (**B**).

To obtain more T_1_ mutants, we sowed the seeds of *osasn2*. However, the seeds of *osasn2* failed to germinate. Even when germinated on the 1/2 MS medium, they still did not germinate (Figure 2). Seeds that appeared to be normally developed still failed to germinate (Figure 2A). The embryo was visible and showed signs of bulging, but it was unable to continue growing. Then, we aimed to obtain homozygotes through the segregation of heterozygotes. However, there was no homozygote in T_1_ generation. Only heterozygous and wild-type plants were germinated.

**Figure 3:**
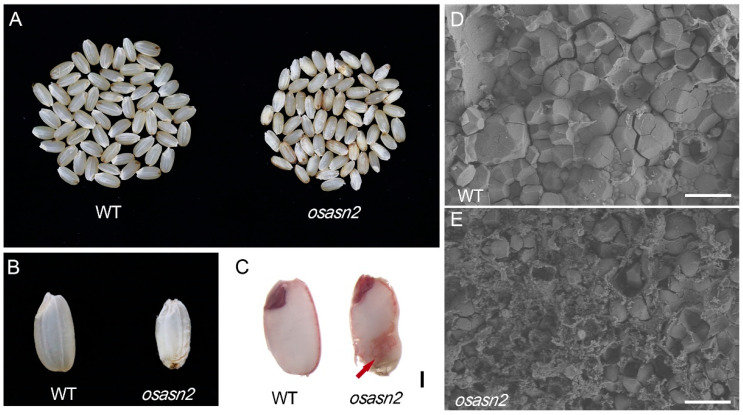
Knockout *OsASN2* resulted in seeds defect. (**A**,**B**) Seeds of *osasn2* were not fully developed. (**C**) Endosperm was defected in *osasn2*, though not the embryo. Seeds were germinated for 12 h and then stained with 0.1% TTC for 2 h. Bar = 100 μm. (**D**,**E**) Scanning electron microscopy (SEM) of WT and *OsASN2* endosperm, respectively. Bar = 10 μm.

We found that a large number of the seeds of *osasn2* were not fully developed, and that the surface of these seeds was not as smooth as the WT (Figure 3A,B). Consequently, we speculated that the embryos of mutants might be impaired. To assess the viability of embryos, the TTC stain was conducted after imbibition. When a seed is soaked in TTC solution, the TTC penetrates the seed tissues, where it accepts a hydrogen ion (resulting from respiration) and forms triphenylformazan. Triphenylformazan is a stable, red-colored, non-diffusible substance. Nonviable seed tissues do not react with TTC and do not stain [23]. The embryos and aleurone cells of *osasn2*, as well as the WT, were stained red, indicating that the embryos and aleurone cells of *osasn2* were as active as those of the WT (Figure 3C). However, abnormal endosperm was observed in *osasn2* (Figure 3C). Then, the endosperm was cross-sectioned and observed by SEM. We found that the starch granules in the WT were neatly arranged and displayed a polyhedral structure. However, the starch granules in the *osasn2* mutants were loosely packed and had cavities lacking starch granules (Figure 3D,E). Quantitative analysis revealed that the average number of starch granules was 6817/mm^2^ in WT, in contrast to only 2861/mm^2^ observed in *OsASN2*.

### 3.2. OsASN2 Was Ubiquitously Expressed in Rice

**Figure 4:**
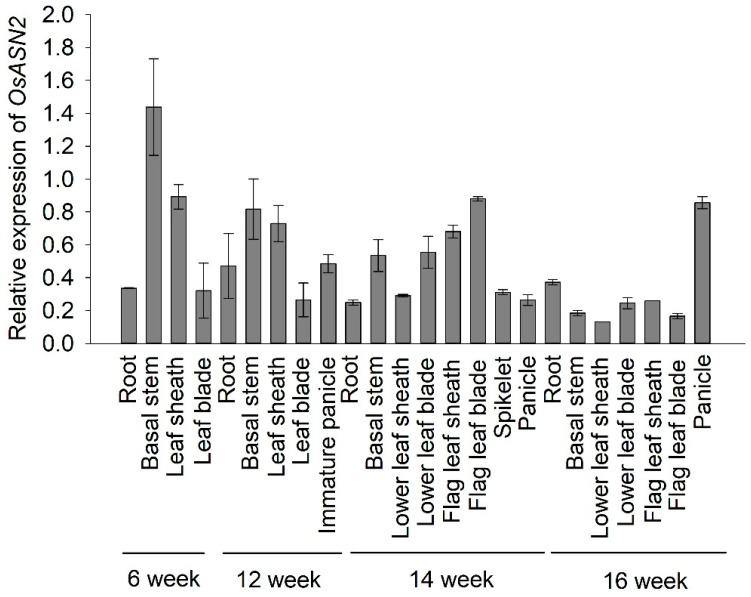
*OsASN2* ubiquitously expressed in rice root, stem, leaf sheath, leaf blade, and panicles at different growth stages. Real-Time RT-PCR (qPCR) analysis of *OsASN2* expression in rice organs at different times after germination.

qPCR analysis revealed that *OsASN2* was ubiquitously expressed in leaves, roots, stems, leaf sheaths, and panicles (Figure 4), suggesting that *OsASN2* plays a role in the entire growth and development of rice. It was noteworthy that *OsASN2* had higher expression levels in the panicles when compared with organs such as the leaf, leaf sheath and stems in the grain-filling stage (week 16 in Figure 4).

### 3.3. OsASN2 Interacted with OsPPDKB

**Figure 5:**
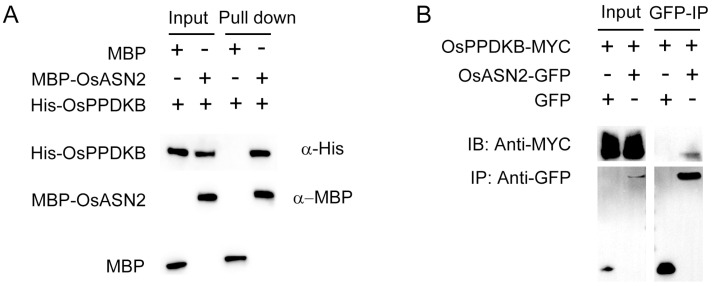
OsASN2 interacts with OsPPDKB. (**A**) In vitro pull-down analysis of OsASN2 and OsPPDKB interaction. Resin-bound MBP-OsASN2 or MBP was incubated with His-OsPPDKB recombinant protein. The elution fractions were detected by an anti-His antibody. (**B**) Co-immunoprecipitation (Co-IP) analysis of OsASN2 and OsPPDKB interaction. GFP or OsASN2-GFP was transiently co-transformed into rice protoplasts with OsPPDKB-MYC, and Co-IP experiments using GFP-Trap were performed 12 h after transformation. Immunoblots were executed with anti-GFP antibodies to detect OsASN2 and with anti-MYC to detect OsPPDKB.

To further investigate the potential proteins OsASN2 interacted with, IP-MS was performed to screen for interaction proteins of OsASN2. This analysis identified 16 specifically enriched proteins, among which OsPPDKB drew our particular attention. PPDK catalyzes the reversible interconversion of pyruvate, phosphate, and phosphoenolpyruvate (PEP) through its dual catalytic activity. *OsPPDKB* knockout mutant also showed defective grain filling by affecting sucrose-to-starch conversion processes [24]. Firstly, an in vitro pull-down assay was performed to check the OsASN2 and OsPPDKB interaction. As in Figure 5A, His-OsPPDKB was pulled down by MBP-OsASN2, but not by MBP alone. Subsequently, Co-IP assay was performed to validate the interaction between OsASN2 and OsPPDKB. As shown in Figure 5B, OsPPDKB-MYC was co-precipitated by OsASN2-GFP, but not by GFP. These results confirm a specific interaction between OsASN2 and OsPPDKB in rice.

## 4. Discussion

Our results suggest that knockout of *OsASN2* has a profound effect on reproductive organs, evidenced by a significant reduction in seed germination (Figure 2), and impaired seed development, particularly endosperm development (Figure 3). These defects in reproductive development coincided with the previous study that *OsASN2* is expressed in the phloem companion cells of leaves and leaf sheaths, and phloem parenchyma cells of young seeds [25]. Additionally, our results reveal that *OsASN2* expressed highly in the grain-filling stages of rice and that it expressed in various organs during all of the development stages (Figure 4). This time–spatial expression is the basis of its role in rice growth and seed development.

Amino acids are crucial throughout a plant’s life. Free amino acids play key roles in the metabolism of developing seeds [18,19]. During seed development, organic nitrogen is transported into the seeds as amino acids, which are incorporated into seed-storage proteins and the tricarboxylic acid (TCA) cycle for carbon metabolism and starch formation. The dense tissue structure of seeds causes internal hypoxia, reducing the efficiency of oxygen-dependent energy production pathways like respiration. Under these conditions, amino acid catabolism through the TCA cycle not only provides ATP but also produces metabolic intermediates that support adaptive energy metabolism networks. This flexibility helps seeds meet developmental needs, especially during the stage of rapid accumulation of storage compounds [26]. In addition to their role in energy metabolism, amino acids serve as precursors for the synthesis of primary and secondary metabolites, including organic acids, osmolytes, phytohormones, and components of the cell wall [27,28]. Consequently, amino acid metabolism plays an even more critical role in developing seeds compared with vegetative tissues [26]. Asp-family amino acids, including Lys, Thr, Met, and Ile, have been shown to be active in seed development, because they are further incorporated into the TCA cycle directly under conditions of energy shortage [29]. Furthermore, levels of Asn and Gln decrease significantly during seed development, much more than other free amino acids. This research suggests that Asn and Gln are primarily utilized for the synthesis of seed-storage proteins and other amino acids derived from their catabolic pathways [30].

The access of amino acids in seed development and seed quality traits has been evidenced by amino acid transporter genes and metabolism enzyme gene function studies. The study of *Lotus japonicus* asparaginase NSE1, catalyzing the conversion of Asn into Asp, indicates that metabolism of Asn and Asp is essential in seed development, as the abnormal seeds were found in mutants [31]. In our study, OsASN2, catalyzing the conversion of Asp into asparagine, also plays critical roles in rice seed development. In contrast, the abnormal seeds of *nse1* had no embryos while *osasn2* had abnormal endosperm. Why this difference exists is an interesting question to be further studied. Regardless, the functional study of these two genes indicates that genes encoding Asn and Asp conversion enzymes play important roles in seed development. The rice amino acid transporter OsAAP6 positively regulates protein content by increasing protein body size, while negatively regulating starch content [32]. Overexpression of maize *ZmAAP6* in the endosperm aleurone layer leads to enlarged protein bodies but reduced starch granules, resulting in decreased endosperm starch content, while the mutant showed the opposite phenotype [33]. These amino acid transporter studies indicate that the amino acid metabolism flux to protein and to starch is tightly regulated, and also indirectly supports our conclusion that *OsASN2* mutation affects starch granule formation. Asn and Gln are thought to be the main amino acids transported in plants. The Gln synthetase gene has been shown to be involved in seed development. Overexpression of the rice glutamine synthetase gene *OsGS1;1b* increases seed length and decreases both protein and amylose contents [34]. In our study, the Asn synthetase gene *OsASN2* also proved to be involved in seed development, particularly starch granule formation, by *osasn2* mutant analysis. An alanine metabolism gene was also shown to be important in seed development. In Fang’s study, mutants of the rice alanine aminotransferase gene *OsAlaAT1* (*lnue1*) produced opaque grains and exhibited lower amylose and total protein contents, greater soluble sugar content, lower grain yield than the wild type [35]. Furthermore, mutation of *OsAlaAT1* also impacted the expression of *OsPPDK*, indicating that amino acid metabolism is tightly related to pyruvate metabolic flux and thus to starch formation. In our study, we found that OsASN2 interacted with OsPPDKB. Our results and Fang’s results both indicate that the *PPDK* might be the target gene regulated by amino acid metabolism in seeds. PPDK in rice and maize is abundantly expressed in the endosperm and is proposed to balance the flow of carbon into starch, protein, fatty acid, and amino acid biosynthetic pathways due to its freely reversible catalysis [36]. The conversion of PEP and Pyr by PPDK might tightly regulate the metabolism of carbon and nitrogen. Due to Asn’s high ratio of C and N, it was thought to be an ideal medium for nitrogen and carbon transport in plants. The interaction between ASN and PPDK may be an important mechanism for the coordinated regulation of carbon and nitrogen during seed development. However, the question of how the interaction affects their respective functions and influences the carbon–nitrogen metabolic flux requires further investigation.

Except for seed development, aspartate family amino acids seem to supply carbon and nitrogen skeletons and energy sources for seed germination [37]. Asp exhibits the highest increase among all kinds of amino acids during *Arabidopsis* germination [30]. Lysine, a product of aspartate metabolism, has been shown to supply substrates for the TCA cycle, thereby generating sufficient energy for both germination and seedling establishment [28]. Cysteine, another product of aspartate metabolism, serves as crucial metabolite during *Arabidopsis* germination [38]. Cysteine not only acts as a block for protein synthesis but also as the precursor of S-adenosylmethionine, which is the precursor for the biosynthesis of polyamines, vitamin biotin, and ethylene. Furthermore, cysteine is fundamental for DNA synthesis, methylation regulation, and hormone regulation. In germinating rice seeds, glutamate was identified as the most abundant amino acid [39]. During seed germination of *Medicago truncatula*, carbon atoms were incorporated into asparagine, aspartate, glutamine and glutamate, which were specifically involved in the biosynthetic pathways of primary amino acids [40]. When treated with ustiloxins A, which has a typical phytotoxicity that inhibits seed germination, most of the amino acids detected in endosperm and embryo were variously decreased, including glutamine, tryptophan, and cysteine [41]. These studies highlight the role of asparagine and glutamine in seed germination from a physiological perspective. Therefore, we propose that genes involved in the transport and catalysis of these amino acids may be critical for seed germination. Recent research supports this hypothesis. Pereira et al. found that *OsAAP1* played an important role in amino acid transport during the early stages of rice development, while knockout *OsAAP1* impaired the initial development of rice seedlings [42]. In Han’s proteomic analysis, glutamate decarboxylase was downregulated during germination [43]. In our study, knockout of *OsASN2* impaired the germination of rice. Germination of *OsASN2* in 1/2 MS medium indicated that the key factor affecting germination might be the defective development of endosperm, because the embryo could be seen to bulge, but did not continue to grow. Even a small portion of the seeds that appeared to be normally developed failed to germinate (Figure 2). Raffan et al. observed similar results in the study of wheat *ASN*, finding that knockout of *TaASN2* in wheat leads to reduced asparagine content in seeds and poor germination [44]. Studies on these functional genes have confirmed the importance of amino acid transport and metabolism genes in seed germination. Among these genes, genes associated with asparagine and aspartate metabolism are of particular significance.

Our results reveal that *OsASN2*, which encodes a primary amino acid metabolism enzyme, contributes to rice seed development and germination. However, the intricate link between amino acid metabolism and reproductive development remains largely unexplored, leaving us with the intriguing question of how *OsASN2* precisely modulates reproductive development. OsASN2 might affect the function of PPDK, thus affecting the carbon metabolism in seed development. Further studies related to carbon and nitrogen metabolism changes and the associated regulation network should be addressed. As the endosperm is critical to grain yield and grain quality, the role of *OsASN2* on grain yield and quality is expected to be evaluated in the future.

## 5. Conclusions

Asparagine is one of the main forms of amino acid transported in plants and has been shown to be related to seed development. The roles of *ASN* genes are worthy of in-depth investigation. In this study, CRISPR/Cas9-mediated knockout mutants of the *OsASN2* gene in rice were generated. Homozygous *OsASN2* exhibited failure of seed germination. Moreover, large numbers of seeds developed abnormally. Further studies have disclosed that the starch granules of *OsASN2* mutants was loosely packed. Additionally, interacting protein screening combined with pull-down and Co-IP assays confirmed that OsASN2 interacted with OsPPDKB. These findings indicate that *OsASN2* is essential for rice seed development and germination, providing insights for a future, detailed investigation into the regulatory mechanisms underlying seed development and germination.

## Data Availability

The original contributions presented in this study are included in the article/Supplementary Materials. Further inquiries can be directed to the corresponding author.

## Supplementary Materials

The following supporting information can be downloaded at: https://www.mdpi.com/article/10.3390/plants14131999/s1.
