# Supplementary figures and images for: Asparagine Synthetase Gene OsASN2 Is Crucial for Rice Seed Development and Germination
Source: Plants (Basel). 2025 Jun 30;14(13):1999. doi: 10.3390/plants14131999 (PMC12251889; doi:10.3390/plants14131999)

Supplemental Figure S1

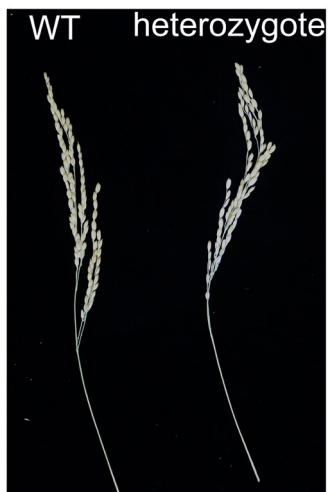

Figure S1 Panicles of heterozygous mutant of *OsASN2* and WT.

Supplement: Supplementary file 1 [file plants-14-01999-s001.zip › plants-3612149-supplementary.pdf]
